# Exploring Future Nursing Leadership Through Student Insights in a Transforming Healthcare Landscape: A Descriptive Study

**DOI:** 10.7759/cureus.91709

**Published:** 2025-09-06

**Authors:** N. Leena Madhura, K.P. Joshi, Deepak C Jamadar

**Affiliations:** 1 Obstetrics and Gynaecology Nursing, SVS College of Nursing, Mahabubnagar, IND; 2 Community Medicine, SVS Medical College, Mahabubnagar, IND; 3 Community Medicine, SVS Medical college, Mahabubnagar, IND

**Keywords:** cross-sectional study, leadership development, mentorship, nursing leadership, nursing students

## Abstract

Objective: This study aimed to assess leadership perceptions, aspirations, and preparedness among final-year nursing students in India, examining the influence of academic level, curriculum support, and mentorship on leadership readiness.

Methods: A cross-sectional, mixed-methods design was employed at SVS College of Nursing, Telangana. The study included 200 final-year students from the Bachelor of Science in Nursing (B.Sc. nursing), general nursing and midwifery (GNM), post-basic B.Sc. nursing, and Master of Science in Nursing (M.Sc. nursing) programs. Quantitative data were collected using a pre-validated, self-administered questionnaire and analyzed using descriptive statistics, chi-square tests, t-tests, and ANOVA. Qualitative data were obtained through four focus group discussions, analyzed thematically using Braun and Clarke’s framework, and triangulated with quantitative findings.

Results: High aspirations for leadership were reported across all programs, with slightly higher levels among B.Sc. and M.Sc. students. However, perceived preparedness was moderate overall, with no statistically significant differences between programs (p > 0.05). Access to mentorship varied but did not significantly correlate with leadership aspirations or confidence. The qualitative findings highlighted barriers such as curriculum gaps, limited role modelling, hierarchical constraints, and the lack of structured leadership exposure during clinical placements.

Conclusion: Final-year nursing students in India show strong motivation to take on leadership roles; however, a noticeable gap remains between their aspirations and perceived readiness. Strengthening curriculum content, enhancing faculty development, improving mentorship systems, and increasing institutional support are essential to developing future nursing leaders.

## Introduction

Nursing has evolved from a profession once limited to bedside care into one that now demands advanced clinical expertise, critical decision-making, and strong leadership capabilities. As modern healthcare systems face mounting challenges, including resource constraints, workforce shortages, and public health crises, nurses increasingly function as frontline leaders, influencing patient outcomes, care quality, and healthcare policy [1,2]. Nursing leadership extends beyond managerial responsibilities to include advocacy, clinical coordination, team management, and quality improvement within healthcare environments [3].

Globally, nurses’ strategic leadership role is well established. The International Council of Nurses (ICN) and the World Health Organization (WHO) have consistently emphasized the importance of investing in leadership development to build robust health systems and achieve universal health coverage, for instance through the WHO’s “Global Strategic Directions for Nursing and Midwifery” (2021-2025), which explicitly identifies leadership as a priority area, and ICN’s “Leadership for Change” program, which trains nurses to influence policy, manage resources, and strengthen workforce capacity [4,5]. These initiatives recommend integrating structured leadership modules into curricula, embedding mentorship, and creating opportunities for clinical leadership practice, offering a model for nursing education and training reforms in India. Recent empirical studies also demonstrate that structured leadership training significantly improves nurses’ self-efficacy, communication skills, and capacity for organizational change [6,7]. However, in India, leadership remains inadequately integrated into nursing education, with curricula emphasizing technical competencies over leadership development [6]. Surveys of Indian nursing schools reveal that less than one-third have formal leadership modules, and students frequently report limited opportunities to practice leadership in clinical rotations [7,8].

Transformational leadership theory, developed by Bass and Avolio, offers a valuable framework for understanding how nursing students’ leadership potential can be nurtured [9]. This theory emphasizes vision, motivation, intellectual stimulation, and ethical practice, qualities that inspire nurses to lead through influence rather than authority [9]. Complementing this, Benner’s Novice-to-Expert Model explains how nurses progressively acquire expertise and leadership skills through experiential learning and clinical judgment. For this study, these frameworks are operationalized by examining final-year students’ self-perceived leadership readiness, confidence in decision-making, and capacity for motivating peers, which align with both transformational leadership qualities and Benner’s “competent-to-proficient” stage [9].

Although leadership competencies are widely acknowledged as valuable [10,11], Indian research highlights persistent structural barriers [6,8,12,13]. Many institutions lack trained faculty, structured mentoring systems, and protected opportunities for leadership practice, leaving students clinically competent but underprepared for leadership roles [12,13]. Mentorship and role modeling have shown promise in bridging this gap [14,15], but Indian nursing schools often face faculty workload constraints and entrenched hierarchies that limit student autonomy [12]. Furthermore, while global evidence suggests that problem-based learning and interprofessional education enhance leadership capacity [16], these pedagogical innovations remain underutilized in India [15].

The National Nursing and Midwifery Commission (NNMC) has recently emphasized the need for competency-based education and leadership development at all levels of nursing education [17]. However, limited empirical research exists on how Indian nursing students themselves perceive their readiness for leadership, the barriers they encounter, and their aspirations across General Nursing and Midwifery (GNM), Bachelor of Science in Nursing (B.Sc.), Post Basic B.Sc., and Master of Science in Nursing (M.Sc.) programs.

The present study investigates final-year nursing students’ perceptions, aspirations, and preparedness for leadership roles across diverse academic programs in India to address this gap. By applying Transformational Leadership Theory and Benner’s Novice-to-Expert Model as guiding frameworks, the study generates actionable insights to inform nursing education reforms and strengthen leadership development in alignment with the evolving demands of India’s healthcare system.

## Materials and methods

Study design, setting, and approach

This study employed a descriptive, cross-sectional survey as the quantitative component, combined with a qualitative focus group strand within a convergent parallel mixed-methods design to examine leadership perceptions and aspirations among final-year nursing students comprehensively. As a quantitative observational approach, the cross-sectional survey collected data from students across different nursing programs at a single point in time. In the convergent design, quantitative and qualitative data were collected during the same phase of the study, analyzed separately, and then integrated to allow for triangulation and comparison [18]. The quantitative strand generated measurable data on demographic characteristics, leadership perceptions, aspirations, and preparedness, while the qualitative strand provided nuanced insights into students’ lived experiences through focus group discussions (FGDs). Integration of findings enabled a richer understanding, with qualitative insights used to explain or contextualize survey patterns. These methods offered both breadth and depth in addressing the research objectives [18].

The study was conducted over three months at SVS College of Nursing, Telangana, India. This institution provides multiple nursing programs, including Bachelor of Science in Nursing (B.Sc. Nursing), General Nursing and Midwifery (GNM), and Post Basic B.Sc. Nursing and Master of Science in Nursing (M.Sc. Nursing), making it an appropriate setting to compare leadership readiness across different academic levels. Data was collected within classroom and group discussion environments on June 20-21, 2025.

Study population and sampling

The study population comprised 200 final-year nursing students: B.Sc. Nursing (n = 100), GNM (n = 60), Post Basic B.Sc. Nursing (n = 30), and M.Sc. Nursing (n = 10). A disproportionate stratified purposive sampling strategy was employed to ensure adequate representation from each nursing program. The strata were defined by academic program: Bachelor of Science in Nursing (B.Sc. Nursing), General Nursing and Midwifery (GNM), Post Basic B.Sc. Nursing, and Master of Science in Nursing (M.Sc. Nursing). This approach was chosen because proportionate sampling would have resulted in very few participants in the Post Basic B.Sc. and M.Sc. strata, limiting comparative analysis. By applying disproportionate stratification, adequate subgroup sizes were maintained while still reflecting the overall distribution of programs within the institution. Lists of eligible final-year students were obtained from departmental records, and students were approached directly during scheduled academic sessions. Participation was voluntary and based on written informed consent after clearly explaining the study’s purpose.

Inclusion and exclusion criteria

Students currently enrolled in the final year of B.Sc., GNM, Post Basic B.Sc., or M.Sc. Nursing programs that provided written informed consent were included in the study. Students on extended leave or those completing clinical internships outside the institution during the study period were excluded, as they were not physically available to participate in classroom-based data collection or focus group discussions. Non-provision of consent was not considered an exclusion criterion, since participation was entirely voluntary by design. Similarly, withdrawal after enrollment was treated as attrition rather than exclusion.

Data collection methods

Quantitative data were collected using a structured, pre-validated, self-administered questionnaire. The tool comprised five sections: demographic profile (age, gender, course, and background); perceptions of nursing leadership roles and responsibilities; leadership aspirations and willingness to assume future leadership positions; perceived enablers and barriers influencing leadership development; and preparedness and support systems, including the effectiveness of curriculum, mentorship, and institutional support. The initial pool of items was developed by reviewing existing international nursing leadership questionnaires and empirical studies, with adaptations to ensure contextual relevance for Indian nursing education. Items were further aligned with constructs from Transformational Leadership Theory and Benner’s Novice-to-Expert Model to provide theoretical grounding. These dimensions reflected the study’s variables of interest, with academic level and mentorship exposure serving as independent factors and perceptions, aspirations, and self-assessed preparedness representing dependent factors.

The questionnaire included multiple-choice items, Likert-scale ratings, and open-ended responses. Content validity was established through expert review by senior nursing faculty, yielding a Content Validity Index (CVI) of 0.87. Reliability was tested using Cronbach’s alpha of 0.82, indicating strong internal consistency. A pilot study involving ten students from a non-participating institution further ensured clarity, reliability, and feasibility. Questionnaires were distributed and collected in classroom settings on the same day. Students completed the questionnaires independently without faculty members present in the room during the response period to avoid any perception of coercion or hierarchical influence. The investigator was available nearby only to clarify procedural queries but did not intervene in the process. Faculty members assisted solely in coordinating logistics (e.g., scheduling classroom access) and were not involved in administering the tool. This approach balanced the need for voluntary participation, as outlined in ICMR ethical guidelines, while ensuring completeness of responses and minimizing non-return of questionnaires.

Qualitative insights were obtained through four FGDs, one for each course group, to complement the quantitative data. FGDs were deliberately chosen over individual interviews or open-ended questionnaires because they facilitate dynamic interaction, allowing participants to build on one another’s experiences, challenge perspectives, and generate richer discussions around leadership, an inherently social and relational concept. While we acknowledge that peer influence can occur in group settings, a trained moderator actively encouraged participation from all members and managed dominant voices to reduce this risk. FGDs also enabled exploration of collective norms and shared challenges that might not emerge in individual accounts. Each group comprised 6-8 purposively selected students (total n = 28) from the quantitative pool, based on their interest and availability. A group-based approach was preferred over one-to-one interviews, as it allowed efficient engagement of a larger number of participants within limited time and resources while still providing depth of insight through group interaction. Discussions lasted 60-75 minutes and were moderated by a trained qualitative researcher using an open-ended discussion guide. Topics included personal leadership aspirations, clinical exposure to leadership roles, role models and mentorship, challenges in developing leadership qualities, and recommendations for institutional improvement. Depending on participants’ preferences, discussions were conducted in English or Telugu. Trained observers documented non-verbal cues, group dynamics, and key quotes in detailed field notes. Audio recordings were made with consent and subsequently transcribed verbatim to preserve accuracy and participant voice. Data saturation was determined when no new themes emerged after the third FGD; one additional FGD was conducted to confirm saturation.

Data analysis

Quantitative data were coded, entered into Microsoft Excel (Redmond, USA), and analyzed using IBM Corp. Released 2017. IBM SPSS Statistics for Windows, Version 26.0. Armonk, NY: IBM Corp. Descriptive statistics were used to summarize demographic and leadership-related variables, including frequencies, percentages, means, and standard deviations. Inferential statistics were applied, including chi-square tests, independent samples t-tests, and ANOVA, to examine associations and group differences. A p-value of < 0.05 was considered statistically significant.

Qualitative data from FGDs were approached using a descriptive qualitative design and transcribed verbatim. The data were then analyzed through reflexive thematic analysis (RTA) following Braun and Clarke’s six-step framework (2006), which emphasizes the researcher’s active role in theme development and iterative engagement with the data. Independent coding by two researchers enhanced reliability, and discrepancies were resolved through discussion until consensus was reached. NVivo software supported data management and organization. Quantitative and qualitative findings were integrated through a triangulation protocol: convergent themes were highlighted as reinforced evidence, while discrepancies were examined to identify contextual explanations (e.g., curriculum variation or institutional culture). In such cases, qualitative insights were used to contextualize quantitative trends, and joint displays were created to show how the two strands informed each other. This process ensured a comprehensive and balanced interpretation of the data.

Ethical considerations

Ethical approval for the study was obtained from the Institutional Ethics Committee of SVC Medical College, Yenugonda, Mahabubnagar, India (Approval No.: IEC/DHR-01/(02/10)/2025/021/10). Written informed consent was obtained from all participants before data collection. Confidentiality and anonymity were maintained by assigning unique participant codes generated by the principal investigator, which were used consistently throughout data handling and analysis. Access to raw data was restricted exclusively to the research team. Participation was voluntary, and students were assured that refusal or withdrawal would not impact their academic standing.

## Results

Table 1 summarizes the mean age and gender distribution of students across different nursing programs. M.Sc. students had the highest mean age at 28.8 ± 2.82 years, followed by B.Sc. students at 24.88 ± 3.23 years, Post B.Sc. students at 24.36 ± 2.78 years, and GNM students at 24.05 ± 3.03 years. In terms of gender, all 100 (100%) B.Sc. students and 60 (100%) GNM students were female. The Post B.Sc. group comprised eight (52%) males and 22 (48%) females, while the M.Sc. group included six (60%) females and four (40%) males.

**Table 1:** Demographic summary by course B.Sc. nursing: Bachelor of science in nursing. GNM: General nursing and midwifery. M.Sc.: Master of science in nursing. Mean age comparison across groups was done using one-way ANOVA (p = 0.06, not significant). Gender distribution across groups was compared using the chi-square test (p < 0.001, statistically significant).

Course	Mean age ± SD	Male N (%)	Female N (%)
B.Sc. (N =100)	24.88 ± 3.23	0 (0%)	100 (100%)
GNM (N = 60)	24.05 ± 3.03	0 (0%)	60 (100%)
Post B.Sc. (N= 30)	24.36 ± 2.78	8 (52%)	22 (48%)
M.Sc. (N= 10)	28.8 ± 2.82	4 (40%)	6 (60%)

**Figure 1:**
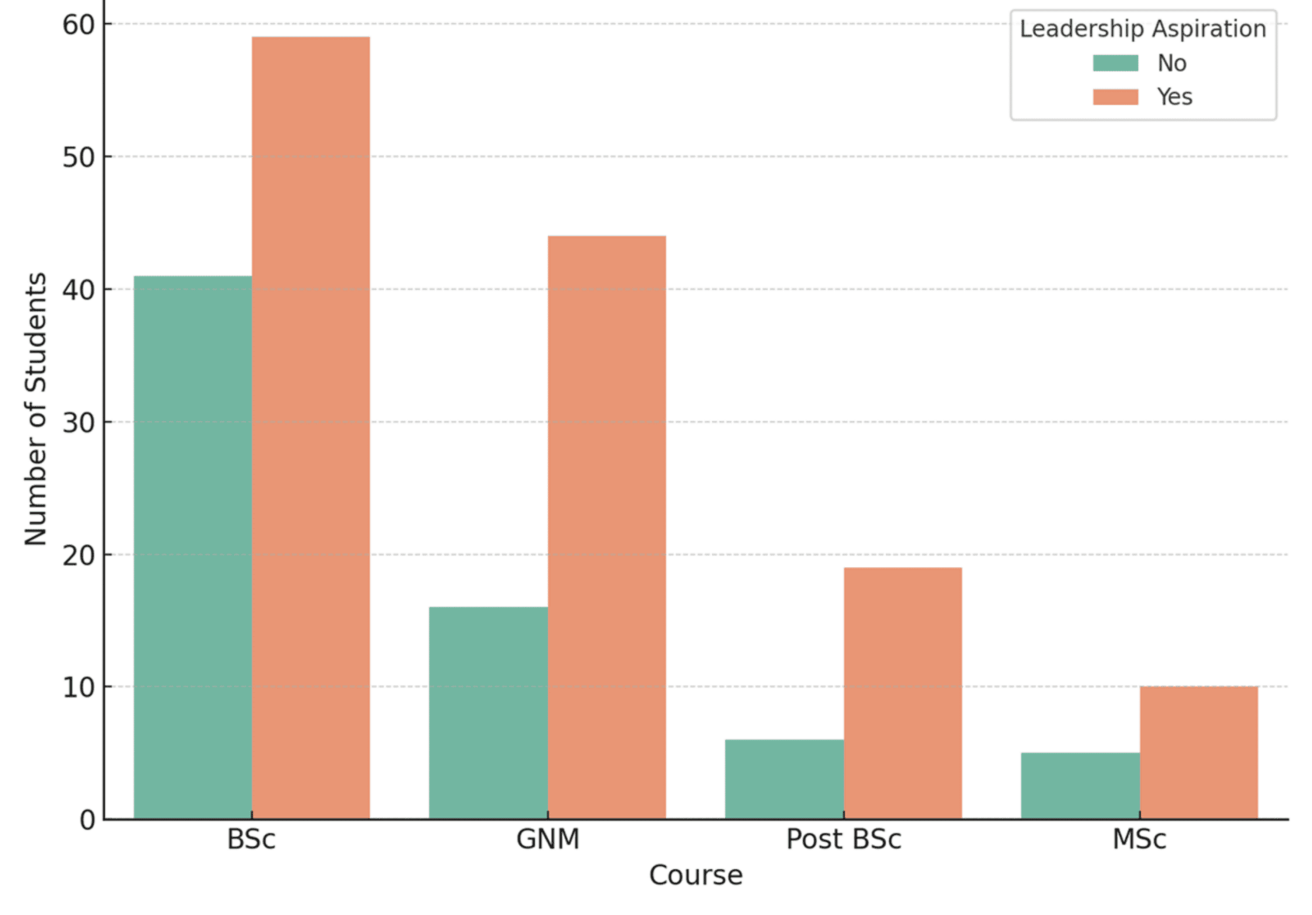
Leadership aspiration by course (%) The image is created by the author.

Figure 1 illustrates the distribution of students across different nursing programs who expressed aspirations for leadership roles. The highest proportion was observed among B.Sc. students, 72 (72%), followed by M.Sc. students, seven (70%), GNM students, 40 (68%), and post-B.Sc. students 19 (63%). A chi-square test of independence showed no statistically significant difference in leadership aspirations across the four programs, χ²(3, N = 200) = 2.12, p = 0.533. The effect size, calculated using Cramér’s V, was 0.103, indicating a small association between course type and leadership aspiration.

**Figure 2:**
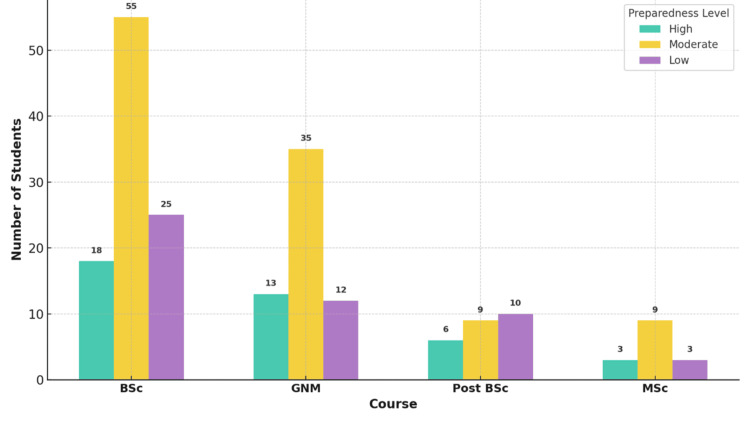
Perceived preparedness by course (%) The image is created by the author.

Figure 2 presents the level of preparedness reported by students for assuming leadership roles. Across all programs, “moderate” preparedness was the most commonly selected category. B.Sc. and M.Sc. students showed slightly higher proportions in the “High” preparedness category, suggesting they may have received more effective leadership training or support. Despite these differences, the chi-square test of independence did not indicate statistical significance, with a chi-square value of 8.62, 6 degrees of freedom, and a p-value of 0.208. The effect size, measured using Cramér’s V, was 0.147, reflecting a small to moderate association between program type and perceived preparedness.

Table 2 presents the distribution of students across different nursing programs based on their responses to leadership aspiration. Among B.Sc. students, 60 (60%) expressed interest in leadership roles, compared to 43 (71.7%) in GNM, 14 (46.7%) in Post B.Sc., and 7 (70%) in M.Sc. programs. A chi-square test of independence was conducted to assess the association between course type and leadership aspiration, yielding a chi-square value of 3.29 with 3 degrees of freedom and a p-value of 0.353. These results indicate no statistically significant difference in leadership aspiration across the programs. The effect size, measured by Cramér’s V, was 0.129. While this value falls within the range typically described as a “small” association, it should be interpreted cautiously, given the lack of statistical significance, and therefore does not provide evidence for a meaningful relationship between program type and leadership aspiration.

**Table 2:** Mentorship access by course B.Sc. nursing: Bachelor of science in nursing, GNM: General nursing and midwifery, M.Sc.: Master of science in nursing.

Course	No N (%)	Yes N (%)	Chi-square value	df	Effect size	p-value
B.Sc. (N =100)	40 (40%)	60 (60%)	3.29	3	0.129	0.353
GNM (N = 60)	17 (28.3%)	43 (71.7%)				
Post B.Sc. (N= 30)	16 (53.3%)	14 (46.7%)				
M.Sc. (N= 10)	3 (30%)	7 (70%)				

**Figure 3:**
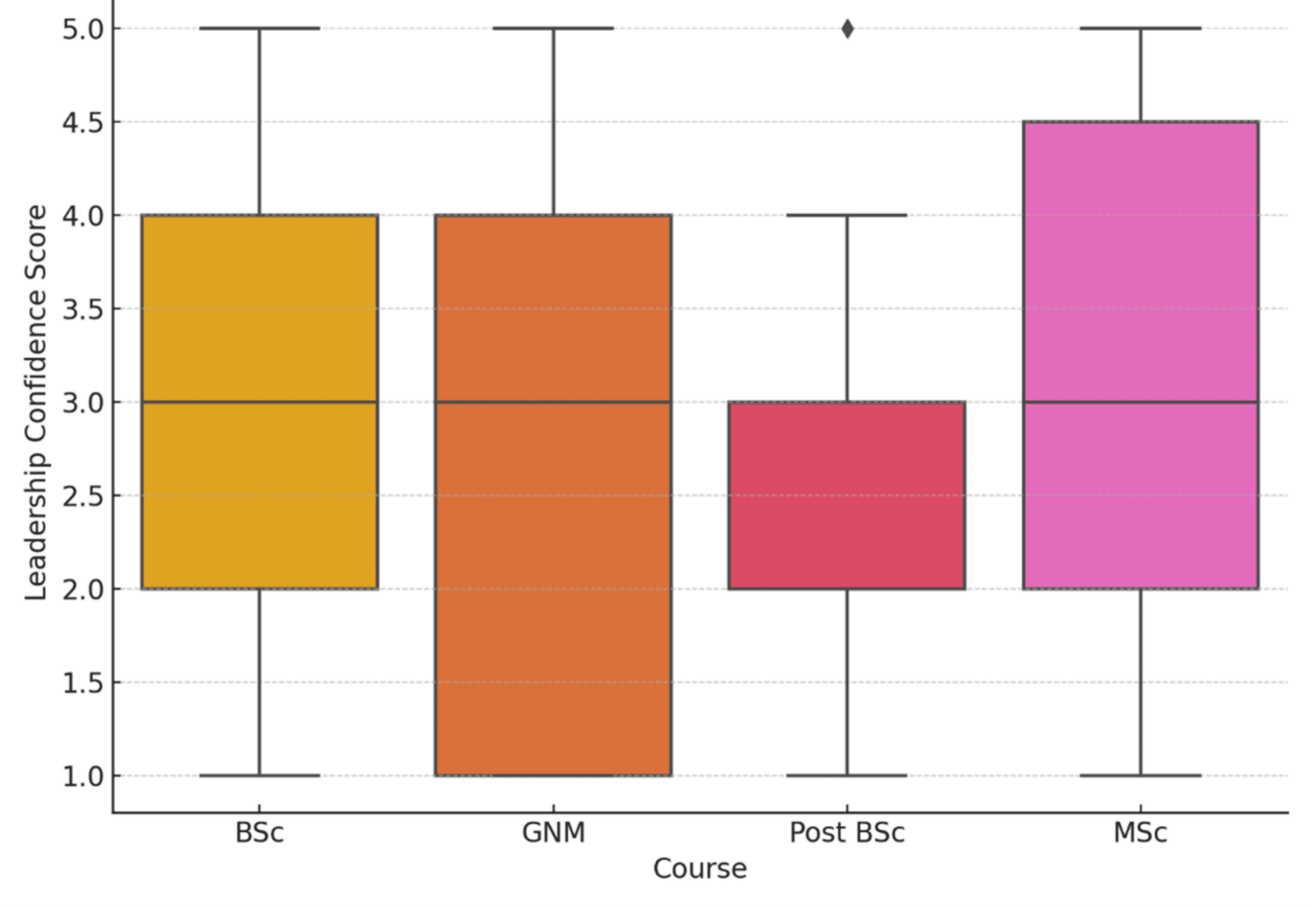
Mean leadership confidence score by course The image is created by the author.

Figure 3 presents leadership confidence scores on a 5-point scale across different nursing programs. The highest mean score was observed among M.Sc. students at 3.13 ± 1.42, followed closely by B.Sc. students at 3.13 ± 1.39. GNM students reported a mean score of 3.02 ± 1.53, while Post B.Sc. students had the lowest average at 2.52 ± 1.31. Although some variation was present, a one-way ANOVA indicated that the differences were not statistically significant, with F = 1.26, p = 0.3022. The effect size, calculated using eta squared, was 0.019, suggesting a small effect of course type on leadership confidence scores.

Table 3 shows the gender distribution of students across different nursing programs. All students in the B.Sc. and GNM programs were female, 100 (100%) and 60 (100%), respectively. In the Post B.Sc. group, 12 (40%) were female and 18 (60%) were male. A chi-square test was conducted to evaluate the association between gender and course type, yielding a chi-square value of 104.69 with 3 degrees of freedom and a p-value of 0.9458, indicating no statistically significant association. The effect size, calculated using Cramér’s V, was 0.72, suggesting a strong association. However, the high V value in the presence of a non-significant p-value may reflect sparse or uneven group sizes affecting test reliability.

**Table 3:** Gender distribution by course B.Sc. nursing: Bachelor of science in nursing, GNM: General nursing and midwifery, M.Sc.: Master of science in nursing.

Course	Female N = 186	Male N =14	Chi-square test	Cramer V effect size	df	p-value
B.Sc. (N =100)	100 (100%)	0 (0%)	104.69	0.72	3	0.9458
GNM (N = 60)	60 (100%)	0 (0%)				
Post B.Sc. (N= 30)	12 (40%)	18 (60%				
M.Sc. (N= 10)	6 (60%)	4 (40%)				

Table 4 shows the relationship between mentorship experience and leadership aspiration among students. Out of 127 students with mentorship, 81 (67.4%) aspired to leadership roles, and 46 (57.4%) did not. Among the 73 students without mentorship, 39 (32.6%) aspired to leadership, and 34 (42.6%) did not. A chi-square test assessed the association between mentorship and leadership aspiration. The test yielded a chi-square value of 1.56 with 1 degree of freedom and a p-value of 0.2112, indicating that the association was not statistically significant. The effect size, measured by Cramér’s V, was 0.088, suggesting a small association between mentorship and leadership aspiration.

**Table 4:** Leadership aspiration vs. mentorship access

	Aspired to leadership	Did not aspire to leadership	Total students	Chi square value	df	Effect size	p-value
Had mentorship	81 (67.4%)	46 (57.4%)	127 (63.5%)	1.56	1	0.088	0.2112
No mentorship	39 (32.6%)	34 (42.6%)	73 (36.5%)				
Total students	120 (100%)	80 (100%)	200 (100%)				

**Figure 4:**
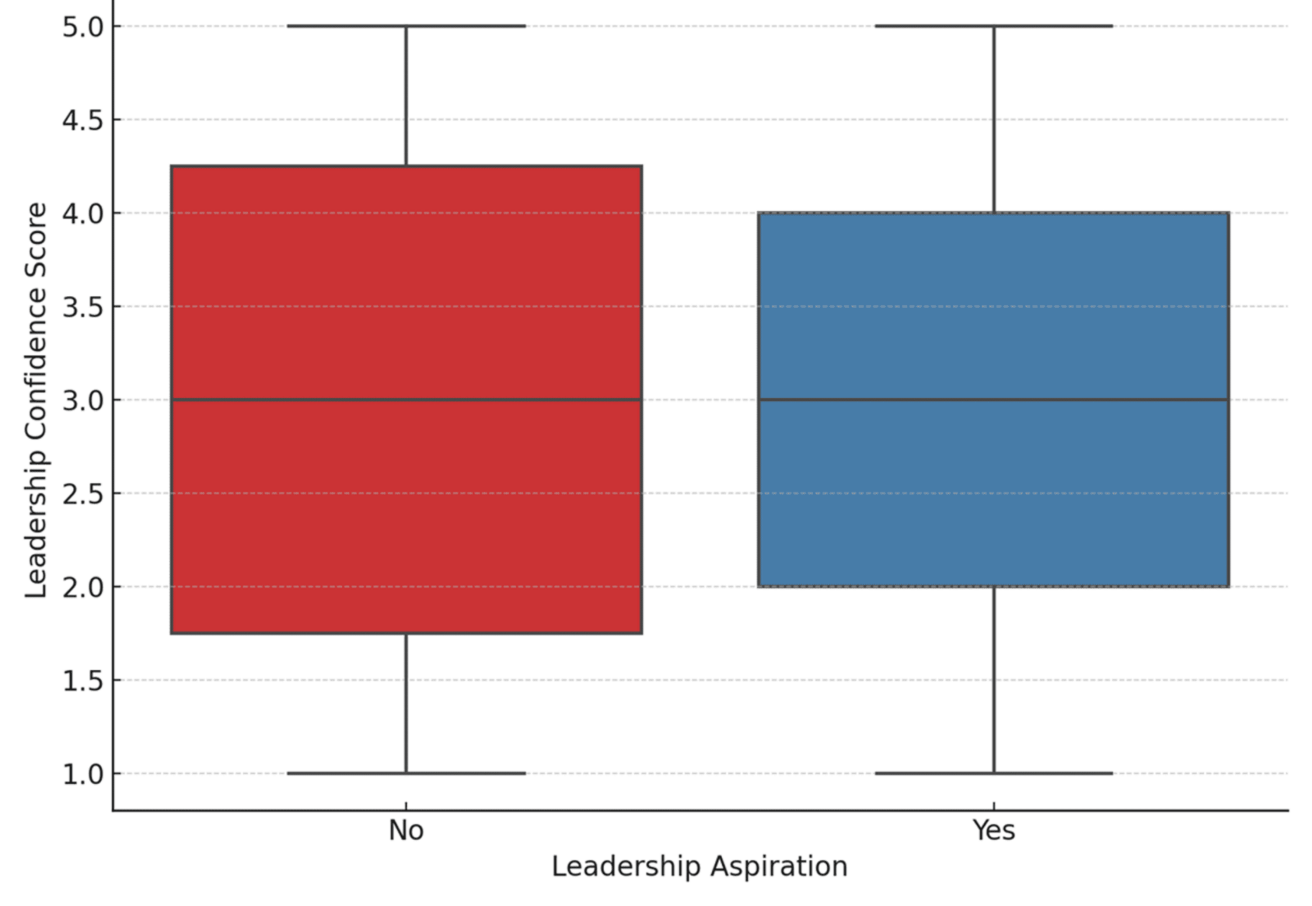
Leadership aspiration vs. leadership confidence score

Figure 4 compares the leadership confidence scores between students who aspired to leadership roles and those who did not. The mean ± standard deviation (SD) for leadership aspirants was 2.99 ± 1.45, while for non-aspirants it was 3.03 ± 1.42, indicating similar levels of self-reported leadership confidence. An independent samples t-test showed no statistically significant difference between the two groups [t(df) = -0.17, p = 0.8655]. The effect size, calculated using Cohen’s d, was 0.03, suggesting a negligible difference in leadership confidence based on aspiration.

Integration and Meta-Inferences

The mixed-methods design of this study enabled a deeper understanding of nursing students’ leadership aspirations by combining survey findings with qualitative insights. Integration of these strands revealed areas of convergence, complementarity, and expansion, producing meta-inferences that strengthen the overall interpretation.

Quantitatively, 124 (71%) of 174 students expressed leadership aspirations, yet only 90 (52%) of 174 reported feeling adequately prepared to take on such roles. This preparedness gap was further illuminated in the qualitative phase, where students consistently described rigid hierarchies during clinical placements, limited autonomy in decision-making, and an institutional emphasis on technical competencies rather than leadership development. The convergence of these results suggests that inadequate preparedness is perceived and structurally reinforced by the training environment.

Mentorship emerged as another critical point of integration. In the survey, 108 (62%) of 174 students reported insufficient mentorship opportunities. The FGDs expanded upon this, revealing that many faculty members were perceived as inaccessible and that deliberate strategies for leadership guidance were lacking. This complementarity shows that insufficient mentorship was less about the absence of faculty per se and more about the lack of structured and intentional mentorship models.

Another important finding relates to the readiness of students to engage in leadership roles. While the majority expressed strong aspiration quantitatively, the qualitative data enriched this by highlighting proactive student-driven recommendations such as curriculum reforms with leadership modules from the first year, structured mentorship frameworks, scenario-based leadership simulations, and greater opportunities for student-led governance and community projects. The integration of these findings points toward a strong motivational foundation among students that institutions can build upon.

By narratively aligning the quantitative counts with qualitative themes, the meta-inferences that emerge are threefold: (1) leadership preparedness is hindered more by systemic and structural barriers than by student reluctance; (2) mentorship capacity-building within faculty is critical to bridge the preparedness gap; and (3) student motivation and concrete recommendations provide fertile ground for implementing reforms. These inferences highlight the shortcomings of current training structures and indicate actionable pathways for institutions seeking to strengthen leadership development in nursing education.

## Discussion

This study offers valuable insights into how final-year nursing students in India perceive, aspire to, and feel prepared for leadership roles in the evolving healthcare context. It highlights the persistent gap between leadership aspirations and actual preparedness, the uneven access to enablers such as mentorship and curriculum support, and the influence of institutional and cultural factors on leadership development.

One of the key findings was that most students, particularly those in B.Sc. and M.Sc. programs, demonstrated high aspirations for assuming leadership roles. This aligns with international evidence that nursing students are generally motivated to take on leadership roles when exposed to supportive academic and clinical environments [1]. Transformational leadership theory suggests that when students observe leadership that is inspiring, intellectually stimulating, and grounded in ethical practice, they are more likely to see themselves as future leaders [14]. However, while leadership aspirations were widespread across all nursing programs, the lack of statistically significant differences between courses suggests that students’ motivation to pursue leadership roles is present regardless of their academic stream. This finding indicates that aspiration alone does not translate into preparedness; without systemic support such as structured mentorship, curriculum integration, and institutional encouragement, students may struggle to convert motivation into leadership readiness.

This gap between aspiration and actual preparedness was notable. Despite strong leadership aspirations, most students rated their preparedness for leadership roles as moderate. B.Sc. and M.Sc. students indicated slightly higher confidence than GNM and post-basic B.Sc. students. Still, these differences did not reach statistical significance (ANOVA p = 0.208), suggesting that a higher academic level alone does not ensure greater leadership readiness. Most students reported only moderate levels of perceived readiness for leadership, echoing previous reports that nursing curricula in India and similar low- and middle-income contexts inadequately address leadership training [6]. Benner’s [9] novice-to-expert model suggests that experiential learning and increasing clinical competence should naturally foster leadership traits. Yet, when real-world leadership opportunities are scarce, students struggle to apply theory to practice.

Curtis et al. [10] emphasized that leadership cannot be effectively developed through lectures alone; it requires deliberate experiential components, mentoring, and structured reflection. In this study, students in the post B.Sc. and GNM streams reported the lowest levels of leadership confidence, likely reflecting shorter or less robust exposure to leadership experiences during their training. This underlines the urgent need to embed leadership development systematically across all academic levels.

Mentorship emerged as a critical but inconsistently available enabler. While around 60-70% of students reported access to mentorship, its impact on leadership aspiration and preparedness was statistically non-significant. This finding is consistent with research showing that informal or poorly structured mentorship programs often fail to deliver their intended benefits [11]. Nowell et al. [18] demonstrated that structured, intentional mentorship significantly enhances students’ professional identity, self-confidence, and readiness for leadership. Similarly, Jayalakshmi et al. [12] found that nurse mentorship in India improved clinical competence and satisfaction but highlighted gaps in standardization. Kane et al. [15] showed that well-designed mentorship interventions positively impact nurse performance in resource-limited settings. Our results highlight the need for formalized, monitored mentorship frameworks tailored to the specific realities of Indian nursing education.

The importance of clinical exposure in building leadership capacity cannot be overstated. Clinical placements should ideally offer students opportunities to observe and practice leadership tasks such as delegation, conflict resolution, and interprofessional collaboration [16]. Yet, many students reported limited decision-making or team leadership involvement during clinical rotations. Kalisch et al. [19] argued that students develop leadership qualities when routinely challenged to solve problems and reflect critically on their practice. Educational strategies such as problem-based learning, interprofessional education, and leadership simulations can bridge this gap but remain underutilized in India’s largely traditional nursing curricula [20].

The institutional climate and faculty capacity significantly impact leadership development. Faculty who serve as positive role models and engage students in reflective leadership discussions help translate theoretical understanding into practice [21]. However, studies reveal that many Indian nursing colleges lack faculty adequately trained to model leadership behaviors or facilitate experiential leadership learning [7]. Baldwin et al. [22] underscored that effective role modelling is integral to shaping students’ leadership identity and professional behaviors. Without faculty capacity-building, leadership education risks remaining a passive theoretical exercise rather than an active developmental process.

Another factor shaping leadership readiness is the entrenched hierarchy within healthcare settings, which often restricts students’ autonomy in decision-making. Fernandopulle [23] highlighted how rigid leadership structures and medical dominance can stifle nurses’ ability to function as leaders at the point of care. Our findings corroborate this concern, with students reporting hesitation to take initiative for fear of making mistakes or challenging established authority. Addressing such cultural barriers requires institutional reform, supportive policies, and active encouragement of nurse autonomy.

Gender norms and societal expectations also affect leadership pathways in nursing. While women dominate the nursing workforce, cultural perceptions of leadership as a male domain can limit encouragement and opportunities for female students to lead [24]. A study by Masibo et al. [25] highlighted how gender stereotypes continue to shape leadership perceptions in nursing globally. Therefore, promoting gender-sensitive leadership training is vital to ensure equitable opportunities for all nursing students.

When comparing our findings with global contexts, it becomes clear that several high-income countries have integrated leadership development into nursing curricula through competency-based training, leadership portfolios, and interprofessional practice [4]. The Indian context remains uneven despite the National Nursing and Midwifery Commission’s push for reforms [26]. Implementation gaps persist due to curriculum rigidity, faculty shortages, and institutional inertia.

Bridging the aspiration-preparedness gap calls for multipronged strategies. Curriculum design must prioritize leadership as a core competency at all levels, supported by practical opportunities such as leadership simulations, peer-led projects, and structured reflection [20]. Faculty development programs should equip educators with the skills to model leadership and provide effective mentorship. In India, initiatives such as the National Health Mission’s capacity-building programs and the Indian Nursing Council’s leadership and management training modules offer structured opportunities to strengthen faculty competencies [7]. Institutions must establish dedicated leadership development cells to organize workshops, invite nurse leaders for guest lectures, and encourage student-led initiatives.

National-level standardized tools to assess leadership competencies, such as leadership-focused Objective Structured Clinical Examinations (OSCEs) or reflective portfolios, can ensure accountability and reinforce learning. Early exposure to policy and governance, through partnerships with health departments and nursing councils, would also broaden students’ perspectives and help align their aspirations with the real-world demands of leadership.

This study identified barriers limiting nursing students’ readiness to assume leadership roles. Similar to prior findings [10,16], participants described rigid hierarchies in clinical settings that restrict autonomy and discourage initiative. Many hesitated to take on leadership tasks, fearing mistakes or criticism from senior staff. The lack of accessible faculty role models further reinforced leadership as a theoretical rather than practical concept, echoing the gap noted by Sharma et al. in India’s nursing curriculum.

Students strongly recommend addressing these barriers by integrating structured leadership modules throughout nursing education, starting at the undergraduate level. They also highlighted the need for consistent, supervised mentorship by trained faculty to model leadership behaviors and build students’ confidence. To translate leadership theory into practice, practical approaches such as scenario-based leadership simulations, active participation in student councils, ward management, and community projects were emphasized. These activities must be complemented by continuous feedback and formal evaluations to monitor progress and ensure sustained improvement in leadership skills. These suggestions are consistent with global recommendations by the WHO [4] and International Council of Nurses (ICN) [5] to embed leadership competencies, such as effective communication, evidence-based decision-making, conflict management, team coordination, critical thinking, advocacy, and ethical practice, within competency-based nursing education frameworks, thereby strengthening the capacity of the nursing workforce to function in complex healthcare environments.

This study’s strength lies in its mixed-methods approach, which triangulated quantitative and qualitative findings to provide a nuanced understanding of the factors shaping leadership development among Indian nursing students. However, its cross-sectional design and focus on final-year students limit the ability to track changes over time or assess early-stage leadership development.

Limitations

This study has certain limitations that should be acknowledged. First, it was conducted in a single institution, which may restrict the transferability of findings to other nursing schools across India with varying curricular frameworks and institutional cultures. Second, the cross-sectional design precludes assessment of leadership perceptions or preparedness changes over time, thereby limiting causal inferences. Third, although self-reported questionnaires and focus group discussions yielded valuable insights, responses may have been influenced by social desirability bias or the perceived expectations of faculty, potentially inflating positive attitudes toward leadership. Fourth, purposive sampling, while appropriate for exploratory inquiry, may have introduced selection bias, as students with a greater interest in leadership might have been more inclined to participate. Fifth, compared to larger undergraduate cohorts, the relatively small subgroup of postgraduate students reduced the statistical power to detect meaningful differences, which may partly explain the non-significant results despite suggestive effect sizes. Finally, while student recommendations were documented in detail, they remain primarily descriptive. Future studies should prioritize and evaluate these recommendations systematically, for example, by testing structured mentorship programs, simulation-based leadership modules, or student governance initiatives, to identify those with the most significant impact and feasibility for integration into nursing curricula.

Future longitudinal research could better capture the evolution of leadership readiness from entry-level training to early professional practice. Such studies should follow nursing students from initial enrollment through graduation and into their early clinical careers, providing insights into how leadership competencies develop, stabilize, or decline in real-world healthcare environments. Long-term tracking would help identify which educational interventions, such as structured mentorship, leadership simulations, and interprofessional practice, have the most sustained impact on building leadership capacity. Comparative studies across institutions and regions could also reveal contextual factors that facilitate or hinder leadership development. This evidence could directly inform curriculum reforms, national competency frameworks, and institutional policies aimed at producing nursing graduates who are not only clinically competent but also equipped to take on leadership roles that drive quality improvement and patient advocacy across India’s evolving healthcare system.

## Conclusions

This study underscores the strong leadership aspirations among final-year nursing students in India, particularly within B.Sc. and M.Sc. programs, while highlighting the gap between motivation and actual preparedness for leadership roles. Although mentorship access and curriculum support were reported, their impact on leadership readiness was inconsistent, reflecting the absence of structured, standardized frameworks. The findings call for integrating dedicated leadership modules, experiential learning opportunities, and faculty-driven mentorship into nursing education. By addressing systemic barriers such as hierarchical constraints and a lack of role models, institutions can foster confident, capable nurse leaders who are prepared to meet the evolving challenges of healthcare.
